# Isoflurane anesthesia impairs the expression of immune neuromodulators in the hippocampus of aged mice

**DOI:** 10.1371/journal.pone.0209283

**Published:** 2018-12-20

**Authors:** Matthew B. Friese, Miriam Nathan, Deborah J. Culley, Gregory Crosby

**Affiliations:** Laboratory for Aging Neuroscience, Department of Anesthesiology, Perioperative and Pain Medicine, Harvard Medical School and Brigham and Women’s Hospital, Boston, MA, United States of America; Imperial College London, UNITED KINGDOM

## Abstract

Cognitive dysfunction is one of the most common postoperative complications experienced by older patients after anesthesia and surgery but the cause remains unknown. Immune molecules are essential for many aspects of neural homeostasis, including learning and memory, and an imbalance in immune neuromodulators is implicated in the development of neural dysfunction. Aging alters the control of neuroinflammatory cascades and general anesthetics are immunosuppressants. Therefore, we hypothesized that general anesthesia disturbs neuroimmune signaling in an age-dependent fashion. We tested this hypothesis by examining gene expression of key immune neuromodulators including IL-1β, TNFα, and CCL2 in the hippocampus of young adult (3 mo) and aged (20 mo) mice following isoflurane anesthesia. We show that isoflurane anesthesia increases expression of these signaling molecules in the hippocampus of young adult mice but decreases it in the hippocampus of old mice. Furthermore, anesthetized old mice had an amplified hippocampal neuroimmune response to systemically administered lipopolysaccharide compared to age-matched carrier controls. Together, these data indicate that isoflurane anesthesia disrupts hippocampal neuroimmune mediator gene expression in the old brain and suggests a potential mechanism by which general anesthesia can contribute to disordered neuronal homeostasis and post-anesthesia cognitive disability in older subjects.

## Introduction

Over 7 million patients 65 years of age or older have surgery annually, often under general anesthesia, and many experience cognitive morbidity such as delirium or postoperative cognitive dysfunction (POCD) [1–4]. These cognitive morbidities are costly emotionally and financially, as affected persons have longer hospitalizations, are less likely to be discharged home after surgery, and have a higher 1-year mortality [2, 5–7]. Despite much work in the area, the etiology of these postoperative cognitive derangements is unclear, with pre-existing cognitive impairment, surgery, and general anesthesia all being implicated [8–10].

General anesthetics have myriad effects on the brain and are also potent immunomodulators and anti-inflammatory agents. In the periphery, commonly used anesthetics such as isoflurane and propofol inhibit macrophage function, suppress endotoxin-induced upregulation of proinflammatory cytokines, and delay clearance of bacteria [11, 12]. The immunomodulatory actions of anesthetics in the CNS are, by contrast, poorly studied and remain a puzzle, especially in the elderly. Studies in young or middle aged adult animals, for example, report no effect of anesthesia either alone or in conjunction with surgery on brain immune responses. In old animals, however, a commonly used volatile general anesthetic administered without surgery increases brain cytokines, while in older humans there appears to be a decrease in neuroinflammation after surgery under general anesthesia [13–16]. As such, the impact of anesthetic agents on expression of immune mediators in the brain in the aged remains enigmatic.

We hypothesized that general anesthesia dysregulates cerebral neuroimmunity in the aged. To test this hypothesis we measured gene expression of the key pro-inflammatory molecules and immune neuromodulators interleukin 1β (IL-1β), tumor necrosis factor α (TNFα), and chemokine (C-C motif) ligand 2 (CCL2) and the anti-inflammatory cytokine interleukin 10 (IL10) in the brains of young adult (3 mo) and aged (20 mo) mice following general anesthesia. We identified a brain region-specific decrease in expression of these signaling molecules after anesthesia but an amplified response to a systemic pro-inflammatory stimulus in old but not young adult mice, suggesting that an anesthesia-induced imbalance in immune signaling could contribute to development of postoperative cognitive impairment in older subjects.

## Materials and methods

### Mice

The Harvard/BWH Institutional Animal Care and Use Committee approved the protocol and all experiments were conducted according to regulations set forth by the Harvard Medical School and Brigham and Women’s Hospital Standing Committee on Animals. Young adult (3 mo) and aged (20 mo) male C57BL/6 mice were purchased from Charles River Laboratory (Wilmington, MA) and housed 4 per cage at constant temperature and humidity with a regular light-dark cycle and had access to both food and water *ad libitum*. Both young adult and aged spontaneously breathing male mice were prospectively randomized to treatment condition and experimental groups were balanced by litter. For anesthesia experiments, mice (N = 5–6 per treatment condition at each time point unless otherwise indicated) were anesthetized with 1.2% isoflurane in 30% oxygen for 4 h, based on previous work in rodents showing that this exposure can lead to cognitive impairment [13, 17–19]. Age-matched control mice were treated identically except that they received 30% oxygen for 4 h. Rectal temperature was monitored and regulated to 37±0.5°C by placing them on a warmed heating pad. In separate experiments, we treated young and aged animals with lipopolysaccharide (LPS 0111:B4, Sigma; St Louis, MO; 500 ug/kg in 1 ml of saline, intraperitoneally, 1 h after induction of anesthesia) to trigger a mild-moderate peripheral and central inflammatory response as described by others [20]. Control mice were treated identically but received an equivalent volume of saline intraperitoneally. All animals were allowed to recover for either 3 or 48 hours after anesthesia and then were anesthetized briefly (< 3 min) and sacrificed by decapitation. The hippocampus and cortex were then removed at 4°C and processed for PCR as described below. We chose 3 h to investigate acute recovery and 48 h because we have demonstrated previously that aged rodents have a working memory deficit at this time whereas young adult animals do not [10, 21].

### Tissue isolation and cDNA synthesis and quantitative PCR

Following decapitation under general anesthesia, cortical and hippocampal tissue from 5–6 animals per treatment condition and time point in microcentrifuge tubes on ice with 1.5ml Buffer RLT (Qiagen, Hoilden, Germany) and disrupted using a Bullet Blender at 4°C (Next Advance, Averill Park, NY). RNA was then isolated using the RNeasy kit following the manufacturers protocol, with DNAse treatment (Qiagen). The RNA was eluted in RNAse free water and the amount of RNA was quantified using a Nanodrop-1000 (ThermoFisher, Waltham, MA). 2.5ug of RNA was then used to make cDNA iScript cDNA synthesis kit (BioRad, Hercules, CA) following the manufacturers protocol, and 3ul from the RT reaction was subsequently used in a 20ul qPCR reaction using the SYBR Green Reaction Kit (Applied Biosystems, Foster City, CA). Please see Table 1 for the sequences of the primers. The qPCR reactions were run on a C1000 Touch Thermocycler (Biorad) per manufacturers protocol. Fold induction was calculated using the 2^-ΔΔCt^ method.

**Table 1:** qPCR primers.

	Forward	Reverse
β-Actin	GTGACGTTGACATCCGTAAAGA	GCCGGACTCATCGTACTCC These appear to be correct for mouse beta actin. Unclear if these are the sequences that were used in the pPCR at this time.
TNFα	CTTCTGTCTACTGAACTTCGGG	CAGGCTTGTCACTCGAATTTTG
IL-10	GCTCTTACTGACTGGCATGAG	CGCAGCTCTAGGAGCATGTG
CCL2	GTCCCTGTCATGCTTCTGG	GCTCTCCAGCCTACTCATTG
IL-1β	ACGGACCCCAAAAGATGAAG	TTCTCCACAGCCACAATGAG

### ELISA

We measured TNFα and CCL2 in the plasma of a separate group of identically treated, age-matched mice (N = 6) following the manufacturers protocol (R&D Systems, Minneapolis, MN). Briefly, blood was collected directly from the left ventricle of the heart at the time of sacrifice and samples placed in EDTA-coated microcentrifuge tubes and centrifuged for 20 min at approximately 1000 × g within 30 min of collection. The plasma was then removed and stored at −20°C until used for measurement. The optical density of each sample determined using a microplate reader (SpectroMax2, Molecular Devices, Sunnyvale, CA) at 450 nm with wavelength correction at 550.

### Statistics

All data are displayed as the mean ± standard deviation. Hippocampal and cortical data were analyzed by 1-way ANOVA followed by Dunnett’s test for correction for multiple comparisons. Plasma data were analyzed by a 2-way ANOVA. All statistical analyses were performed using GraphPad Prism version 6.

## Results

All animals survived anesthesia and were included. Temperature was maintained within the target range and mean arterial blood pressure (82 ± 13 mmHg) and blood gases (pH 7.36 ± 0.02, PaCO2 47.4 ± 5, PaO2 475 ± 43 mmHg), measured in a separate group of identically anesthetized old mice prepared with a femoral arterial line, were within the physiologic range at the end of anesthesia.

### Isoflurane decreases gene expression of immune signaling molecules in the hippocampus of aged but not young mice and increases expression of IL-10

**Fig 1:**
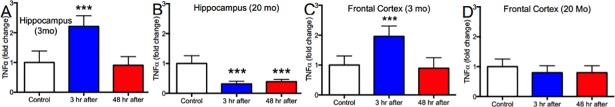
Isoflurane produces age- and region-dependent changes in TNFα gene expression. Mice were anesthetized as described and TNFα measured in the hippocampus and frontal cortex by RT-PCR. TNFα increased transiently in the hippocampus and frontal cortex of young isoflurane anesthetized mice but, like IL-1β, was persistently reduced in the hippocampus but not frontal cortex of old mice demonstrating age and regions specific effects of treatment with isoflurane on TNFα expression. Data expressed as mean ± SD for N = 6 animals per treatment condition. *** p<0.001 relative to age-matched controls by 1-way ANOVA with Dunnett’s test.

**Fig 2:**
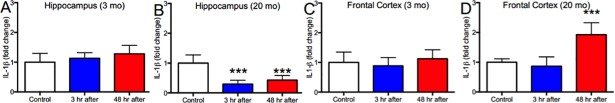
Isoflurane produces age- and region-dependent changes in IL-1β gene expression. 3 and 20-month-old mice were anesthetized with 1.2% isoflurane in 30% oxygen for 4 h and sacrificed 3 h or 48 h later and IL-1β measured in the hippocampus and frontal cortex by RT-PCR. Isoflurane had no effect in either region in young mice (panels A & C). In contrast, it produced a marked and sustained decrease in IL-1β mRNA in the hippocampus and an increase in the cortex 2 d later in aged mice (panels B & C) demonstrating age and region specific effects of treatment with isoflurane. Data expressed as mean ± SD for N = 6 animals per treatment condition. *** p<0.001 relative to age-matched controls by 1-way ANOVA with Dunnett’s test for multiple comparisons.

**Fig 3:**
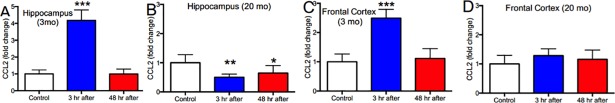
Isoflurane produces age- and region-dependent changes in CCL2 expression. Mice were anesthetized as described and CCL2 measured by RT-PCR. CCL2 was upregulated transiently in both regions in young mice after anesthesia (panels A & C). In old mice, however, isoflurane had no effect in the cortex (D) but persistently decreased CCL2 in the hippocampus (panel B). Data expressed as mean ± SD; N = 5 animals per treatment condition. * p<0.05, ** p<0.01, *** p<0.001 relative to age-matched controls by 1-way ANOVA with Dunnett’s test.

Confirming previous studies, we found age-dependent differences in the levels of immune neuromodulators, with expression of IL-1β and CCL2 being 2–4 fold greater in the hippocampus of aged compared to young controls whereas IL10 is ~50% lower (Table 2). We likewise found that age differentially affects the neuroimmune response to isoflurane anesthesia. In young mice, hippocampal TNFα increased after anesthesia (p<0.001, N = 6; Fig 1) and returned to baseline 48 h later but there was no change in IL-1β at either recovery time (p>0.05, N = 6; Fig 2). Similar changes occurred in the frontal cortex of young mice and the pattern was not specific to cytokines because CCL2, a chemokine implicated in pro- and anti-inflammatory responses and neuroplasticity, also increased 3 h after anesthesia in both the hippocampus and cortex (p<0.001, N = 5; Fig 3) but returned to baseline 48 h later.

**Table 2:** TNFα, CCL2 and IL-10 gene expression in the hippocampus of control 3 m old (young) and 20 m old (aged) mice.

Gene	Young (3 mo)	Aged (20 mo)
TNFα	1 ± 0.53	4.49 ± 1.27*** These appear to be correct for mouse beta actin. Unclear if these are the sequences that were used in the pPCR at this time.
CCL2	1 ± 0.28	2.56 ± 1.69***
IL-10	1 ± 0.42	0.49 ± 0.43*

Expression of the proinflammatory genes TNFα and CCL2 was substantially higher in old compared to young mice and expression of the anti-inflammatory cytokine IL-10 was lower. Data expressed as mean ± SD and analyzed using a Student’s t-test. N = 5–6 animals per treatment condition.

* p<0.05 and

*** p<0.001.

**Fig 4:**
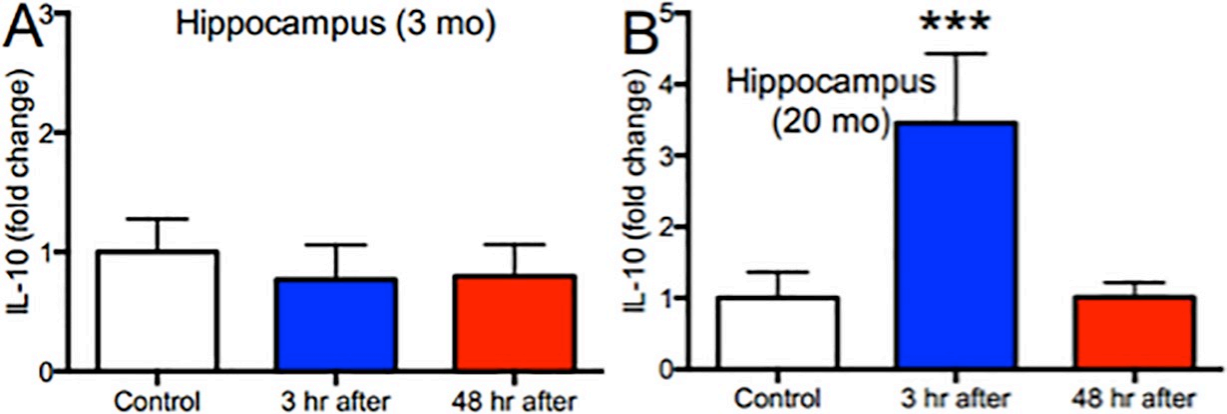
Isoflurane increases IL-10 expression in the hippocampus of aged but not young mice. Mice were anesthetized as described and IL-10 measured in the hippocampus by RT-PCR. Isoflurane had no effect in either region in young mice (A) but increased IL-10 nearly 4-fold in old mice. Data expressed as mean ± SD for N = 6 animals per treatment condition. *** p<0.001 relative to age-matched controls by 1-way ANOVA with Dunnett’s test for multiple comparisons.

**Fig 5:**
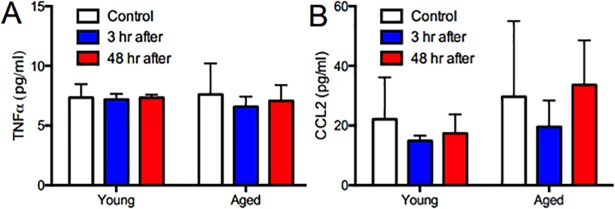
Isoflurane has no effect on plasma TNFα or CCL2 in young or aged mice. Mice were anesthetized as above and TNFα and CCL2 measured in plasma 3 and 48 h later by ELISA. There was no effect of age (p>0.05), treatment condition (p>0.05), or any interactions between the two (p>0.05). Data expressed as mean ± SD for N = 5–6 animals per treatment condition and analyzed using a 2-way ANOVA.

In contrast, in aged animals, IL-1β (p<0.001, N = 6), TNFα (p<0.001, N = 6), and CCL2 (p<0.001, N = 5) decreased in the hippocampus such that expression 3 h after anesthesia was only ~30–50% of the corresponding unanesthetized control level (Figs 1–3). Moreover, expression remained low for at least 48 h (Figs 1–3). Also, unlike in the young adult mice, the frontal cortex of old mice was relatively spared, with the only change in this region being an increase in IL-1β expression 48 h after anesthesia (p<0.001, N = 6; Fig 1). These changes do not represent global depression of hippocampal cytokine or chemokine expression in aged animals because IL10 increased nearly 4-fold transiently (p<0.001, N = 6; Fig 4), a change not observed in young mice (p>0.05, N = 6; Fig 4). Moreover, these effects are independent of circulating inflammatory mediators, as plasma levels of TNFα and CCL2 were unchanged following anesthesia in both age groups (p<0.05, N = 5–6; Fig 5).

### Isoflurane amplifies the neuroinflammatory response to a peripheral inflammatory stimulus in old but not young mice

**Fig 6:**
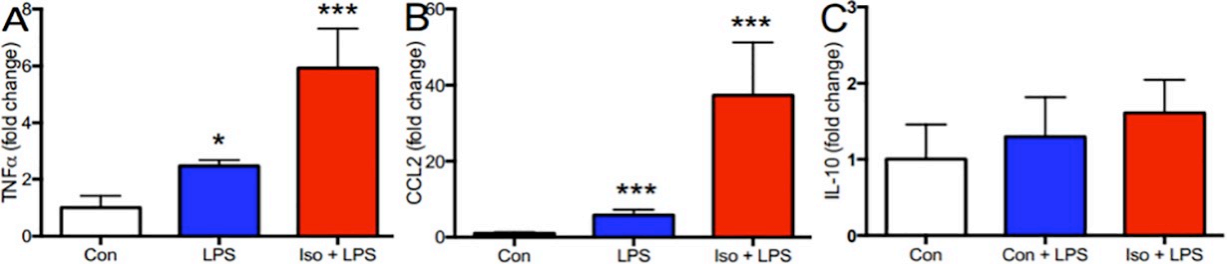
Isoflurane amplifies the hippocampal neuroinflammatory response to LPS in old mice. 20 month old mice were anesthetized as described and 1 h later received an intraperitoneal injection of LPS (500 ug/kg). Control mice received 30% oxygen plus an equal volume of saline i.p. or LPS. Anesthesia continued for 3 h (4 h total) to mimic ongoing surgery under general anesthesia; controls received 30% oxygen for the same period. Mice were sacrificed 3 h after emergence and TNFα, CCL2 1, and IL10 expression measured in the hippocampus by RT-PCR. As expected, LPS alone increased TNF (~3-fold) and CCL2 (~6-fold). This response was markedly exaggerated in old mice that received anesthesia, with TNF increasing ~6-fold and CCL2 up ~37-fold. Levels of IL-10 were slightly and non-significantly increased after LPS and LPS irrespective of isoflurane exposure (C). Note differences in the y-axis scale. Data expressed as mean ± SD for N = 5–6 animals per treatment condition. * p<0.05, *** p<0.001 by 1-way ANOVA with Dunnett’s test for multiple comparisons.

Given the immune neuromodulator hypoactivity we observed after anesthesia in the hippocampus of aged mice, we questioned whether isoflurane anesthesia might also attenuate the neuroimmune response to a peripheral inflammatory stimulus. To investigate that possibility, we administered LPS intraperitoneally to aged animals during isoflurane anesthesia. As expected, LPS markedly increased TNFα(p<0.05, N = 5–6; Fig 6A) and CCL2 transcription (p<0.001, N = 5–6; Fig 6B) in the hippocampus of unanesthetized old mice. To our surprise, however, the hippocampal TNFα and CCL2 responses to LPS were greater still when the mice received LPS while anesthetized (6- and 37-fold increase in TNFα and CCL2, respectively, over the unanesthetized control; p<0.001, N = 5–6). In contrast, LPS had no effect on IL10 regardless of anesthesia (p>0.05, N = 5–6; Fig 6C).

## Discussion

We demonstrate that isoflurane anesthesia leads to marked and enduring suppression of IL-1β, TNFα, and CCL2 expression in the hippocampus, but not frontal cortex, of aged mice, but amplifies the acute hippocampal TNFα and CCL2 responses to peripheral LPS. In young adult mice, by contrast, isoflurane anesthesia increases TNFα and CCL2 expression in the hippocampus and frontal cortex, an effect that resolves by 48 h, and has no effect on IL-1β. While isoflurane anesthesia does not cause suppression of TNFα and CCL2 in the cortex of old animals, as it does in the hippocampus, the transient induction of these genes is no longer observed as in young animals. This anesthetic-induced down-regulation of neuroimmune signaling molecules in the aged hippocampus, and lack of induction in the cortex, is remarkable because the levels of these mediators is substantially higher at baseline than they are in the young brain. These dual effects of isoflurane consisting of suppression of immune mediators under resting conditions and compromised regulation of the neuroimmune response to systemic inflammation are consistent with an age-dependent anesthetic-induced disruption of intrinsic homeostatic mechanisms of neuroimmune signaling and control. If these effects are additive or synergistic in the setting of surgery, they may contribute to the slow or incomplete cognitive recovery reported in older subjects postoperatively. Indeed, in previous work, we have demonstrated, and others have confirmed, a defect in hippocampus-dependent learning and memory 48 h after isoflurane anesthesia in aged but not young animals [10, 13].

Long associated largely with disease and memory loss, neuroinflammation is a double-edged sword. Uncontrolled or chronic overexpression of proinflammatory mediators such as IL-1β, TNFα, and CCL2, as occurs in CNS diseases and after injury, is associated with memory loss and can be harmful, impede recovery, and exacerbate cellular dysfunction [22–24]. However, many of the proinflammatory mediators have functions independent of inflammation, and are important neuromodulators that mediate neural homeostasis and synaptic plasticity in the central nervous system. This concept of molecular pleiotropy, where one protein has multiple functions in different contexts [25], is now well-established, particularly in relation to neuroplasticity. For instance, glial release of the cytokine TNFα is required for homeostatic synaptic scaling, a process by which synaptic strength is altered to compensate for perturbations in neuronal electrical activity [26]. Similar effects on memory and synaptic function and integrity have been reported for IL-1β, CCL2, and IL10 [27–30]. General anesthetics such as isoflurane potently suppress neural activity [31], and thus it is possible that increased levels of TNFα and other immune mediators following anesthesia, as we observed in young mice, are required for restoration of neural homeostasis and cognitive function after general anesthesia, whereas a lack thereof may be associated with delayed or incomplete recovery.

Along similar lines, our data indicate isoflurane general anesthesia perturbs the hippocampal neuroinflammatory response to systemic inflammation. Specifically, despite suppressing hippocampal expression of proinflammatory mediators under resting conditions, isoflurane markedly amplified the TNFα and CCL2 responses of this region to LPS, without affecting IL10. This provides further evidence that isoflurane disrupts endogenous homeostatic processes and immune cascades in the old brain and is an important observation given that general anesthetics are typically used in the setting of systemic inflammation (i.e. surgery). It also supports the hypothesis that dysregulated neuroimmune mediator balance and signaling after general anesthesia and surgery may contribute to the greater vulnerability of older subjects to cognitive dysfunction postoperatively.

Only one study has directly examined the impact of general anesthesia and surgery on brain immune responses in humans [16]. The study was small and used positron emission tomographic (PET) imaging of [11C]PBR28, a marker of glial cell activation, to directly investigate neuroinflammation in older patients after abdominal prostatectomy surgery. The results suggest the neuroimmune system is hypoactive in older patients after surgery, not hyperactive as many previous animal and blood and cerebrospinal fluid biomarker studies have reported. This CNS immune suppression was associated with cognitive impairment, though the sample size was small and testing was limited. That result is similar to what we found in mice with isoflurane alone focusing on key neuromodulators and inflammatory mediators, though none are glia-specific. On the other hand, we found augmented expression of immune molecule transcripts with the combination of LPS and isoflurane anesthesia, which is inconsistent with the human study. There are several potential explanations for this discrepancy including species differences and time of sampling but the most obvious one is the difference in the nature of the proinflammatory stimulus: surgery produces sterile inflammation whereas LPS is a bacterial product. While it is clearly necessary to reproduce our results in a surgical model of inflammation, the human data support the general premise that persistent suppression of immune signaling in the brain may play an important role in the genesis of postoperative cognitive impairment.

This study has several limitations. First, we did not assess animals for neurobehavioral deficits. However, an association is expected because in previous work using similar anesthetic conditions (dose and time) [13, 17–19] we and others have demonstrated impairment in hippocampus-dependent memory in aged but not young adult rodents after anesthesia with isoflurane [10, 21]. The fact that we used only a single anesthetic agent at a single dose and duration limits generalizability to other anesthetic conditions. Nor did we age-adjust the dose of isoflurane, meaning the aged animals received a slightly higher effective dose. However, this is unlikely to explain the differences we observed because changes in the aged were the opposite of those in the young rather than an accentuation of them. We also used only male mice, so the results may not be generalizable to females because there are differences between the sexes in immune cell density and function [32]. The results could be non-specific effects of isoflurane due to altered systemic physiology or circulating inflammatory mediators but this is unlikely because we found different responses between ages, brain regions, and immune molecules, systemic physiology was largely normal, and plasma TNFα and CCL2 levels were unaffected by isoflurane anesthesia. Another limitation is that we have no evidence for immune hypoactivity at the cellular level, such as by changes in microglial morphology. However, as morphological changes are not a reliable measure of microglial function, we have studies underway to examine the impact of anesthesia on the microglial transcriptome. Finally, although we have demonstrated isoflurane anesthesia is associated with an age-dependent, sustained decrease in expression of immune mediators in the hippocampus, more work involving manipulation of immune cascades and concurrent behavioral assessments is necessary to prove that an imbalance in immune cascades causes postanesthetic cognitive impairment.

## Conclusion

Our data demonstrate that isoflurane anesthesia turns down the hippocampal neuroimmune system in old mice and weakens its ability to keep the central response to a peripheral inflammatory stimulus in check. This anesthesia-induced dysregulation of neuroimmunity is enduring and represents a potential mechanism by which general anesthesia may delay or impair cognitive recovery in older subjects.

## Supporting information

S1 Dataset Primary_data. (XLSX)
